# Fully-automated deep learning-based flow quantification of 2D CINE phase contrast MRI

**DOI:** 10.1007/s00330-022-09179-3

**Published:** 2022-10-29

**Authors:** Maurice Pradella, Michael B. Scott, Muhammad Omer, Seth K. Hill, Lisette Lockhart, Xin Yi, Alborz Amir-Khalili, Alireza Sojoudi, Bradley D. Allen, Ryan Avery, Michael Markl

**Affiliations:** 1 Department of Radiology, Northwestern University, 737 N Michigan Ave, Suite 1600, Chicago, IL 60611 USA; 2 Department of Radiology, University Hospital Basel, University of Basel, Petersgraben 4, 4031 Basel, Switzerland; 3 Circle Cardiovascular Imaging Inc., 800 5th Avenue SW, Suite 1100, Calgary, AB Canada

**Keywords:** Blood flow, Blood flow velocity, Deep learning, Pulmonary artery, Magnetic resonance imaging

## Abstract

**Objectives:**

Time-resolved, 2D-phase-contrast MRI (2D-CINE-PC-MRI) enables in vivo blood flow analysis. However, accurate vessel contour delineation (VCD) is required to achieve reliable results. We sought to evaluate manual analysis (MA) compared to the performance of a deep learning (DL) application for fully-automated VCD and flow quantification and corrected semi-automated analysis (corSAA).

**Methods:**

We included 97 consecutive patients (age = 52.9 ± 16 years, 41 female) with 2D-CINE-PC-MRI imaging on 1.5T MRI systems at sinotubular junction (STJ), and 28/97 also received 2D-CINE-PC at main pulmonary artery (PA). A cardiovascular radiologist performed MA (reference) and corSAA (built-in tool) in commercial software for all cardiac time frames (median: 20, total contours per analysis: 2358 STJ, 680 PA). DL-analysis automatically performed VCD, followed by net flow (NF) and peak velocity (PV) quantification. Contours were compared using Dice similarity coefficients (DSC). Discrepant cases (> ± 10 mL or > ± 10 cm/s) were reviewed in detail.

**Results:**

DL was successfully applied to 97% (121/125) of the 2D-CINE-PC-MRI series (STJ: 95/97, 98%, PA: 26/28, 93%). Compared to MA, mean DSC were 0.91 ± 0.02 (DL), 0.94 ± 0.02 (corSAA) at STJ, and 0.85 ± 0.08 (DL), 0.93 ± 0.02 (corSAA) at PA; this indicated good to excellent DL-performance. Flow quantification revealed similar NF at STJ (*p* = 0.48) and PA (*p* > 0.05) between methods while PV assessment was significantly different (STJ: *p* < 0.001, PA: *p* = 0.04). A detailed review showed noisy voxels in MA and corSAA impacted PV results. Overall, DL analysis compared to human assessments was accurate in 113/121 (93.4%) cases.

**Conclusions:**

Fully-automated DL-analysis of 2D-CINE-PC-MRI provided flow quantification at STJ and PA at expert level in > 93% of cases with results being available instantaneously.

**Key Points:**

*• Deep learning performed flow quantification on clinical 2D-CINE-PC series at the sinotubular junction and pulmonary artery at the expert level in > 93% of cases.*

*• Location detection and contouring of the vessel boundaries were performed fully-automatic with results being available instantaneously compared to human assessments which approximately takes three minutes per location.*

*• The evaluated tool indicates usability in daily practice.*

**Supplementary Information:**

The online version contains supplementary material available at 10.1007/s00330-022-09179-3.

## Objectives

2D time-resolved (2D-CINE) phase contrast (PC) MRI (2D-CINE-PC-MRI) was first developed in the 1980s and is today’s clinical standard for in vivo blood flow analysis with MRI [1–3]. 2D-CINE-PC-MRI can be used for blood flow analysis in any larger vessel but mainly the aorta and the main pulmonary artery (PA) [3, 4]. To achieve reliable results, delineation of the vessel contours for each time point of the cardiac cycle is required. Due to dynamically changing vessel lumen contours and movement throughout the cardiac cycle, accurate quantification of flow parameters requires careful delineation of the vessel boundaries [2]. Currently, commercially available software supports radiologists by applying semi-automatic workflows for vessel contour delineation. However, this still requires manual input and is cumbersome; time-consuming manual correction and contour review by an experienced observer may be necessary [5].

Deep learning (DL) is an advanced artificial intelligence (AI) technique which has recently been applied to cardiac MRI, for example, automated left ventricular (LV) segmentation based on cine imaging data to evaluate LV function or in aortic anatomy segmentation [6–8]. For 2D-CINE-PC-MRI applications, Bratt et al described a method for flow assessment using deep learning on clinical series, but it was limited to a single location (aorta) and net flow was the only parameter reported [9]. In this study, we evaluated the performance of a DL tool which automatically performs vessel detection, contouring, and flow quantification from 2D-CINE-PC-MRI data of the aorta and PA. Thereby, it reports both flow volume as well as flow velocity parameters. Our aim was to investigate whether DL results were comparable to manual and semi-automated human flow quantification in a cohort of patients undergoing standard clinical 2D-CINE-PC-MRI, including an assessment of human inter-observer variability.

## Methods

### Study cohort

We included consecutive patients who underwent standard-of-care cardiothoracic MRI including 2D-CINE-PC-MRI series in November 2020. Patients were enrolled retrospectively with a waiver of consent. All patients (*n* = 97) received 2D-CINE-PC at the sinotubular junction (STJ). A subgroup of 28/97 patients (28.9%) also received 2D-CINE-PC at the main PA. This study was approved by the institutional review board, and the need for informed consent was waived.

### MRI acquisition

All exams were performed on 1.5-T MRI systems (Aera, Avanto or Sola, Siemens Healthcare). In general, a 3-chamber (3CH) 2D-CINE-PC series was used to determine the measurement plane of the through-plane series at the STJ which was planned to be perpendicular to the blood flow axis. Alternatively, other 3CH series (scouts or cine series) were used. For the PA location, two in-plane cine images of the PA were acquired. These were used for through-plane plane placement. Velocity encoding (VENC) was set to 150 cm/s as a standard for both locations, if higher velocities were suspected, it was either set to 300 cm/s or alternatively a VENC scout was acquired.

MRI parameters (mean): TE/TR: 3.1 ms/47.1 ms, flip angle: 20°, spatial resolution: 1.8 mm × 1.8 mm, slice thickness: 6 mm, matrix: 192 × 150, VENC: 160 cm/s, number of cardiac time frames acquired: 20 (range: 20-30). 2D-CINE-PC series were always acquired prior to potential contrast application.

### Human flow analysis

**Fig. 1:**
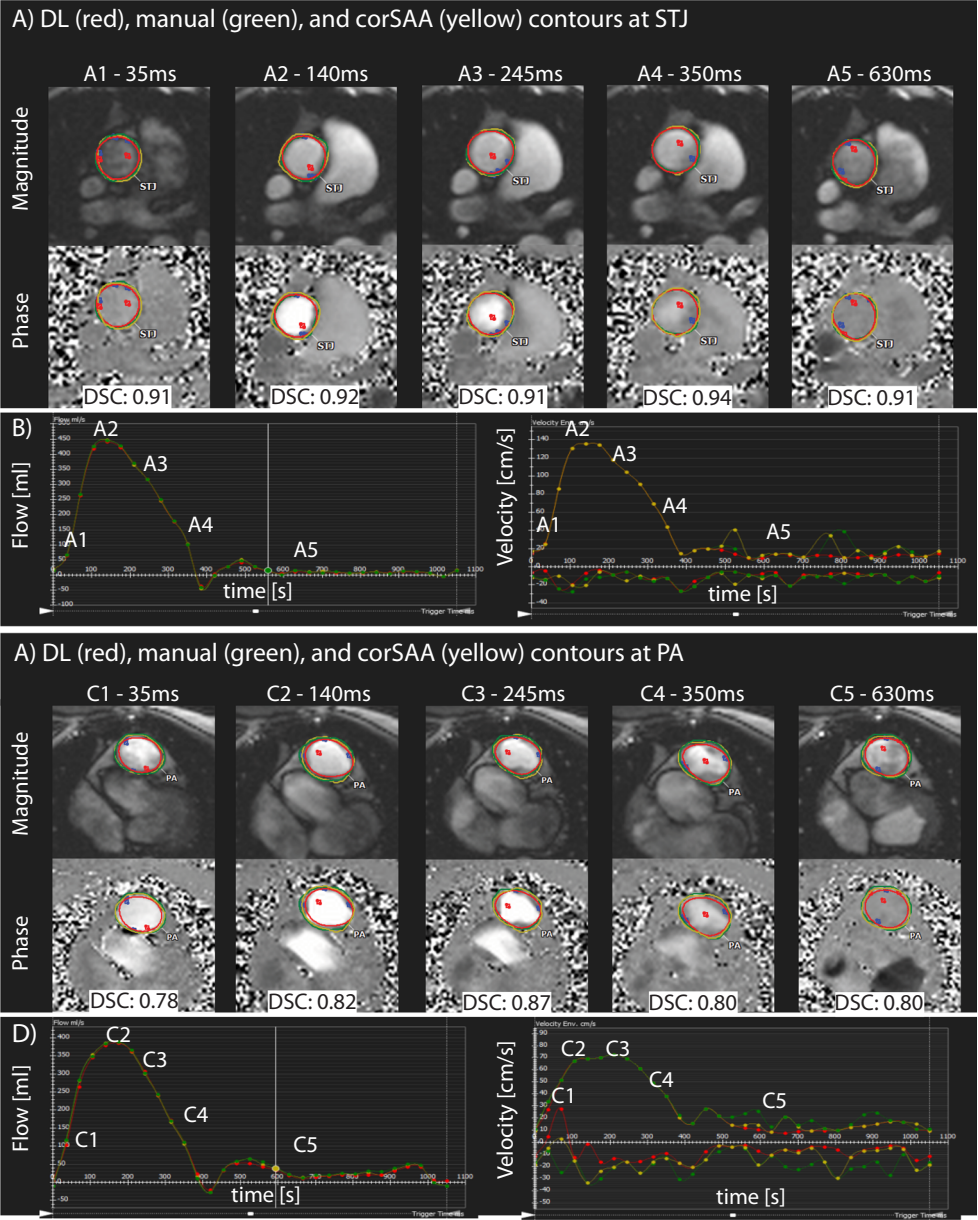
Overview measurements. Demonstration of human (manual analysis (MA) and corrected semi-automated analysis (corSAA)) and deep learning (DL) measurements in one case for five time points with respective volume-over-time and velocity-over-time curves, each showing the time points measured. **A**, **B** Measurements at STJ. (A1-5) Magnitude (top) and phase contrast images for STJ measurements with respective DSC between MA (green) and DL contours (red) for each time point. **B** Flow volume and flow velocity curves at STJ. **C**, **D** Measurement in PA. (C1-5) Magnitude and phase contrast (PC) images for PA measurements with respective DSC between MA (green) and DL contours (red) for each time point. **D** Flow volume and flow velocity curves at PA. corSAA, corrected semi-automatic analysis; DL, deep learning; DSC, Dice score; MA, manual analysis; PA, pulmonary artery; PC, phase contrast; STJ, Sinotubular junction

Analysis was performed using commercial software (cvi42, Circle Cardiovascular Imaging Inc.) by a CV-radiologist with 3 years of experience (M.P.). Three different readings were performed, manual analysis (MA), uncorrected semi-automated analysis (uncorSAA) and corrected semi-automated analysis (corSAA) (Fig. 1). We considered MA the reference because it was unbiased regarding vessel contouring compared to the semi-automated approach.

For MA, the vessel contour was delineated for the first time point on the magnitude images. Then, the contour was copied and manually adjusted on the next time point; this process was repeated for all time points and the time required was recorded. For uncorSAA, the vessel was contoured on the first time point of the magnitude images, this contour was forwarded and adjusted by the software. Of note, this was not a DL technique. The corSAA was based on the uncorSAA contours, but the radiologist checked the contour at each time point and adjusted it if necessary. The total time for checking and adjusting was recorded. Subsequent flow quantification for each approach included the calculation of net, forward, and backward flow. In addition, peak velocity was automatically calculated based on the voxel with the highest velocity during systole, defined as the first third of all time points available.

### Deep learning tool

**Fig. 2:**
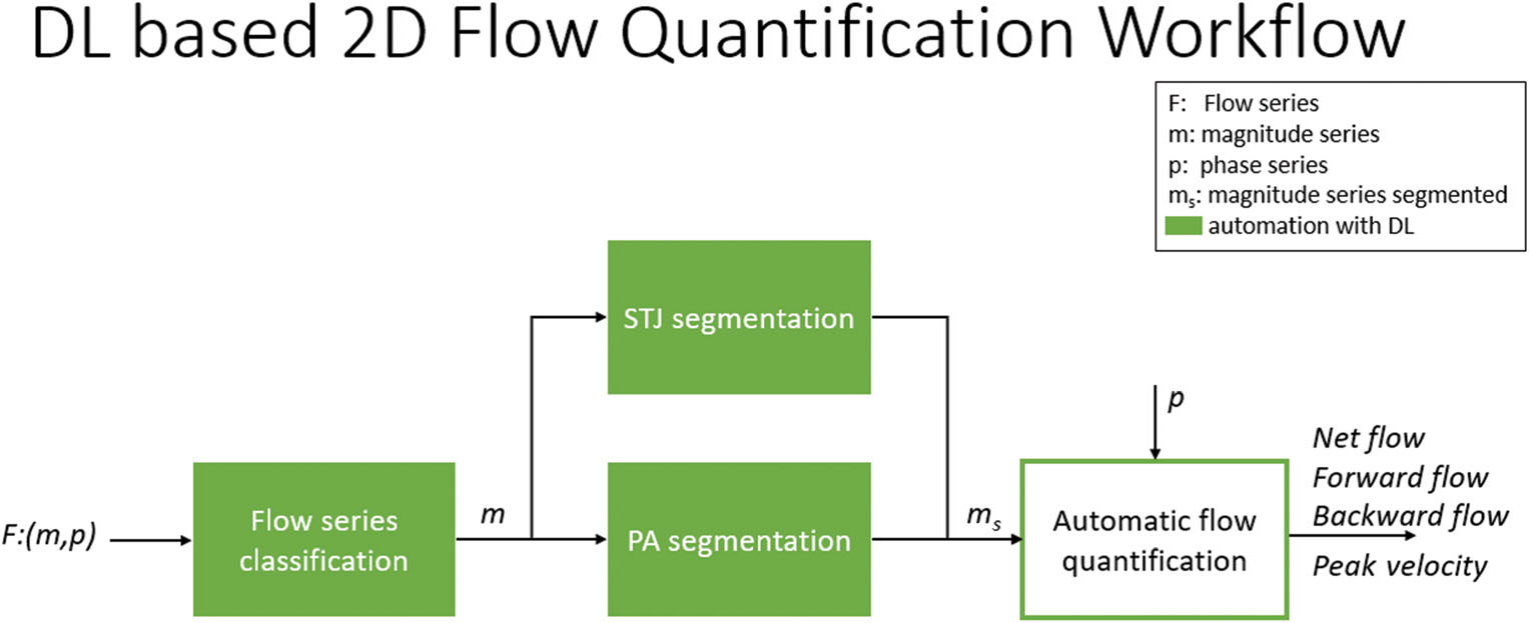
DL-based Flow Quantification Workflow. First, DL identified the location of the images (STJ or PA). Then, location-specific DL contouring was performed on the magnitude images which were used for fully-automatic flow and velocity quantification from the phase series. DL, deep learning; PA, pulmonary artery; STJ, sinotubular junction

The proposed method of automated 2D-CINE-PC-MRI flow quantification involved two stages of automation using deep convolutional neural networks (DCNN, Fig. 2); a detailed description can be found in the supplements. Briefly, the first stage performed a series classification task to identify the input flow series type (aorta or PA). Next, depending on the type of flow series, corresponding segmentation models using a U-Net style architecture were applied to the flow magnitude images to contour the vessel boundaries [10]. A normalization layer was inserted before the U-Net and a inputs were resampled using a special transformer [11, 12]. The output of the segmentation model paired with the flow phase images was input to the subsequent flow quantification stage to compute flow parameters.

#### DL analysis workflow

DL analysis was performed in cvi42. Vessel contours and flow parameters were automatically available using a built-in batch processing tool which created all measurements including visual outputs. Analysis time for each case was noted automatically. If a case could not be processed, no DL results were available which was defined as failure. All cases were processed a second time after 4 weeks by the DL tool to investigate DL variability.

### Comparisons between human vs. DL-based 2D-CINE-PC-MRI analyses

We compared the human (MA, uncorSAA, corSAA) and DL-derived contours by calculating Dice similarity coefficient (DSC) as well as Hausdorff distances (HD) for each cardiac time point and slice location. To further evaluate the accuracy of automatic contours for different cardiac phases, a subgroup analysis between MA and DL was performed for the first third (defined as systole) and the remaining cardiac time points (defined as diastole).

Accuracy was defined as less than ± 10 mL net flow or ± 10 cm/s peak velocity difference between human and DL assessment. Cases with differences above these cut-offs were subsequently reviewed in detail.

### Inter-observer comparison

We randomly selected 10 cases with STJ and PA series available for inter-observer assessment. Those cases were independently contoured by a 2^nd^ year radiology resident (S.K.H.) who had previously completed a CV rotation. The reader was blinded to all other results. Results between the readers’ MA were compared.

### Statistical analysis

Statistical analysis was performed in Python (Python Software Foundation) [13]. First, data was tested for normal distribution. Flow parameters between the three human and DL results were compared with repeated measures ANOVA, in case of significance, we performed posthoc analyses with paired *t-*tests between groups using Bonferroni correction. We also performed a pairwise analysis of the four methods for flow quantification using Pearson correlation, scatter plots, and Bland-Altman analysis to quantify bias (mean difference) and limits of agreement (LoA). In general, a *p* value of < 0.05 was considered statistically significant.

## Results

### Study cohort

We included 97 patients in this study (41 female (42.3%), mean age 52.9 years (range: 25–87 years)). The primary indications were aortic aneurysm/dilatation (known and suspected, *n* = 41), aortic valve disease (*n* = 32), bicuspid aortic valve (*n* = 26), status post aortic surgery (*n* = 15, 3 congenital heart disease cases), and other (*n* = 9). See Table 1.

**Table 1:** Baseline parameters

Number of patients	97
Female sex [number] (%)	41 (42.3%)
Age, mean ± SD	52.9 ± 16 years
BMI, mean ± SD	24.4 ± 5.1 kg/m^2^
Total number of 2D-CINE-PC scans	125
Number of STJ series	97 (77.6%)
Number of PA series	28 (22.4%)

Baseline characteristics of the patient cohort. *BMI* body mass index, *PA* pulmonary artery, *SD* standard deviation, *STJ* sinotubular junction

### DL vs human analysis workflow

Examples of DL and human (MA and corSAA) contours at STJ and PA are demonstrated in Fig. 1. Human analyses (MA, uncorSA, corSA) were successfully completed for all 125 2D-CINE-PC-MRI data (100%). DL was successfully applied to 97% (121/125) of cases (STJ: 95/97 (98%), PA: 26/28 (93%)). In four of the processed 121 cases, the location detection mixed STJ and PA locations. All cases were reprocessed with the DL tool after a 4-week period, the processed case numbers, contours, and measurements were identical, representing zero variability. In total, after a detailed review (please see chapter below), the DL tool performed accurate measurements in 113/121 cases (93.4%).

### DL performance for vessel contour delineation

Compared to MA as the reference, the highest DSC scores at STJ were found for corSAA (mean DSC ± standard deviation (SD): 0.94 ± 0.02, mean HD ± SD: 1.5 ± 0.4 mm), followed by DL analysis (DSC: 0.91 ± 0.02, HD: 1.9 ± 0.5 mm). DL vs. corSAA also had a high agreement (DSC: 0.92 ± 0.02, HD: 1.5 ± 0.5 mm). At PA, the highest agreement was also found between MA and corSAA (DSC: 0.93 ± 0.02, HD: 1.4 ± 0.4 mm) while MA vs. DL analysis showed still high but overall lower performance than at STJ (DSC: 0.85 ± 0.08, HD: 2.1 ± 0.9 mm).

On the other hand, uncorSAA showed lower performance at both locations compared to MA (at STJ, DSC: 0.89 ± 0.06, HD: 2.6 ± 1.2 mm; at PA, DSC: 0.88 ± 0.06, HD: 2.3 ± 1.1 mm); especially HD was the highest at each location indicating worse performance. Of note, DSC scores between uncorSAA and corSAA were high (at STJ, DSC: 0.93 ± 0.05; at PA, DSC: 0.92 ± 0.06); however, these results were biased because of uncorrected contours (for example on the first time frame). This resulted in a DSC of 1.0 per time point, leading to a biased, false-high agreement. A detailed figure comparing DSC and HD between groups can be found in the supplements (Supplement Figure 2).

Comparing systole and diastole, mean DSC and HD were similar at STJ (DSC: 0.90 ± 0.08 vs. 0.90 ± 0.09, HD: 2.2 ± 1.7 mm vs. 2.3 ± 2.2 mm). At PA, DL performance was better during systole compared to that during diastole (DSC: 0.86 ± 0.11 vs. 0.82 ± 0.13, HD: 3.5 ± 5.0 mm vs. 3.7 ± 4.8 mm).

### Flow quantification

#### Flow volume analysis

Detailed results can be found in Table 2. At STJ, the net flow was similar between the four different assessment methods (*p* = 0.48, bias: − 0.9 mL–0.0 mL). However, we observed significant differences for the other parameters: Forward flow was overall significantly different between techniques (*p* = 0.02). Posthoc tests revealed that compared to MA, DL-analysis reported significantly lower values (bias = 1.8 mL, *p* = 0.01). DL analysis vs. corSAA assessment was not significantly different (*p* = 0.06). Assessment of backward flow also revealed significant differences between groups (*p* < 0.001). In post hoc tests, DL-analysis and corSAA were significantly lower than MA but the bias was less than 1 mL (DL vs. MA: bias = − 0.9 mL, *p* < 0.001; MA vs. corSAA: bias = − 0.5 mL, *p* = 0.003).

**Table 2:** Flow parameters at STJ and PA

	Manual analysis (reference)	DL analysis	Uncorrected Semi-automated analysis	Corrected Semi-automated analysis	*p* value
*Sinotubular junction*					
Net flow	88.2 ± 31.6 mL	87.0 ± 31.3 mL	88.1 ± 31.7 mL	88.2 ± 30.9 mL	0.48
Pearson r vs. MA	-	0.97 (*p *< 0.001)	0.99 (*p *< 0.001)	1.00 (*p *< 0.001)	
Bias (Limits of agreement)	-	0.9 mL (− 13.2–15.0 mL)	0.1 mL (− 9.6–9.7 mL)	0.0 mL (− 4.5–4.6 mL)	
*p* value		n.s.	n.s.	n.s.	
Forward flow	95.6 ± 30.4 mL	93.6 ± 30.8 mL	95.4 ± 30.7 mL	95.1 ± 30.1 mL	**0.02**
Pearson *r* vs. MA	-	0.98 (*p *< 0.001)	0.99 (*p *< 0.001)	1.00 (*p *< 0.001)	
Bias (Limits of agreement)	-	1.8 mL (− 11.5–15.1 mL)	0.3 mL (− 9.2–9.5 mL)	0.5 mL (− 4.0–5.0 mL)	
*p* value		**0.01**	0.46	0.03	
Backward flow	− 7.4 ± 12.4 mL	− 6.6 ± 11.7 mL	− 7.2 ± 12.6 mL	− 7.0 ± 11.7 mL	**< 0.001**
Pearson r vs. MA	-	0.99 (*p *< 0.001)	0.99 (*p *< 0.001)	0.99 (*p *< 0.001)	
Bias (Limits of agreement)	-	− 0.9 mL (− 4.4–2.5 mL)	− 0.2 mL (− 3.9–3.5 mL)	− 0.5 mL (− 3.4–2.5 mL)	
*p* value		**< 0.001**	0.24	**0.003**	
Peak velocity	163.4 ± 60.6 cm/s	151.7 ± 57.8 cm/s	161.6 ± 62.6 cm/s	153.1 ± 58.0 cm/s	**< 0.001**
Pearson *r* vs. MA	-	0.85 (*p *< 0.001)	0.93 (*p *< 0.001)	0.90 (*p *< 0.001)	
Bias (Limits of agreement)	-	12.8 cm/s (− 52.4–78.1 cm/s)	1.8 cm/s (− 41.9–45.5. cm/s)	10.3 cm/s (− 41.9–62.4 cm/s)	
*p* value		**< 0.001**	0.42	**< 0.001**	
*Pulmonary Artery*					
Net flow	86.7 ± 29.4 mL	87.0 ± 27.6 mL	87.0 ± 28.9 mL	85.7 ± 28.4 mL	0.051
Pearson *r* vs. MA	-	0.99 (*p *< 0.001)	0.99 (*p *< 0.001)	1.00 (*p *< 0.001)	
Bias (Limits of agreement)	-	1.8 mL (−7.2–10.8 mL)	− 0.3 mL (−10.1–9.4 mL)	1.0 mL (− 4.8–6.7 mL)	
*p* value		n.s.	n.s.	n.s.	
Forward flow	90.9 ± 26.5 mL	90.1 ± 25.9 mL	90.9 ± 26.1 mL	89.8 ± 25.5 mL	**0.008**
Pearson *r* vs. MA	-	0.99 (*p *< 0.001)	0.98 (*p *< 0.001)	0.99 (*p *< 0.001)	
Bias (Limits of agreement)	-	2.4 mL (− 6.0–10.9 mL)	0.0 mL (− 9.4–9.5 mL)	1.1 mL (− 4.8–7.0 mL)	
*p* value		**0.009**	0.89	0.04	
Backward flow	−4.2 ± 6.8 mL	− 3.1 ± 5.7 mL	− 3.9 ± 7.0	− 4.1 ± 6.8	**0.006**
Pearson r vs. MA	-	0.99 (*p *< 0.001)	0.99 (*p *< 0.001)	1.00 (*p *< 0.001)	
Bias (Limits of agreement)	-	− 0.6 mL (− 2.9–1.7 mL)	− 0.4 mL (− 2.0–1.3 mL)	− 0.1 mL (−1.0–0.8 mL)	
*p* value		**0.01**	0.07	0.39	
Peak velocity	110.7 ± 62.7 cm/s	88.6 ± 51.9 cm/s	112.9 ± 60.8	106.5 ± 61.4	**0.04**
Pearson r vs. MA	-	0.89 (*p *< 0.001)	0.84 (*p *< 0.001)	0.96 (*p *< 0.001)	
Bias (Limits of agreement)	-	11.0 cm/s (− 37.1–59.1 cm/s)	− 2.3 cm/s (− 69.4–64.9 cm/s)	4.1 cm/s (− 29.5–37.8 cm/s)	
*p* value		0.03	0.62	0.33	

This table shows the performance of each technique compared to MA (reference). At STJ, the net flow was not significantly different between human and DL assessments. However, forward and backward flow and peak velocity showed small but significant differences resulting in DL analysis and reporting lower values than MA. At PA, the net flow was also not significantly different but forward flow, backward flow, and peak velocity were significantly different between assessments

Numbers are mean ± standard deviation. Significant *p* values are in boldface; the *p* value threshold for each pairwise comparison to being significant was < 0.013 due to the Bonferroni correction. *DL* deep learning, *PA* pulmonary artery, *STJ* sinotubular junction

**Fig. 3:**
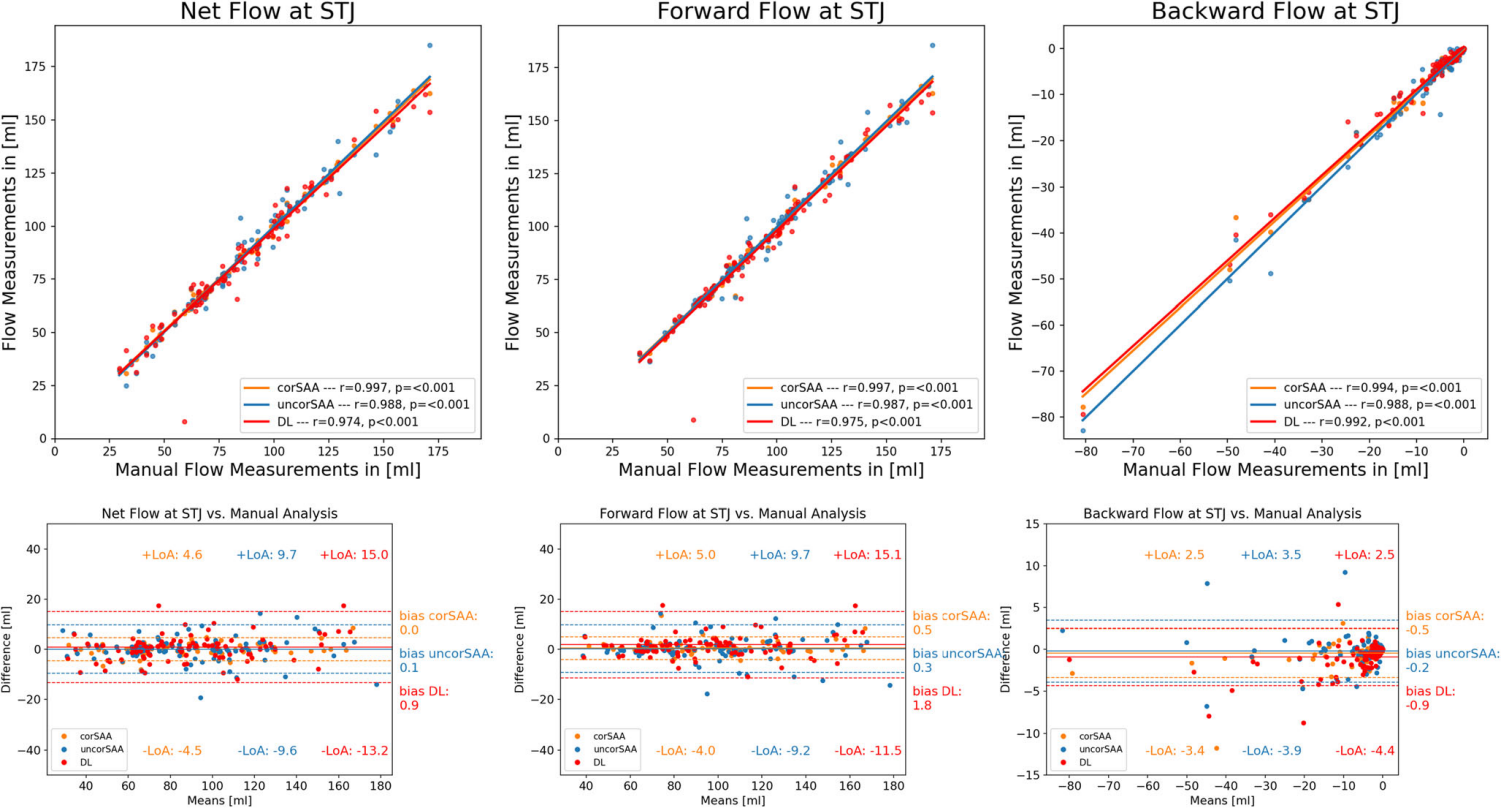
Flow volume measurements at STJ. Comparison of net flow, forward flow, and backward flow at STJ between manual analysis (MA, reference) with corrected semi-automated analysis (corSAA, orange), uncorrected semi-automated analysis (uncorSAA, blue), and deep learning (DL, red) analysis. Scatter plots (top) show excellent correlation and Bland-Altman plots (bottom) overall small bias with narrow limits of agreement between human and DL measurements for each parameter which were not significantly different for net flow assessment. corSAA, corrected semi-automated analysis; DL, deep learning; STJ, sinotubular junction; uncorSAA, uncorrected semi-automated analysis

**Fig. 4:**
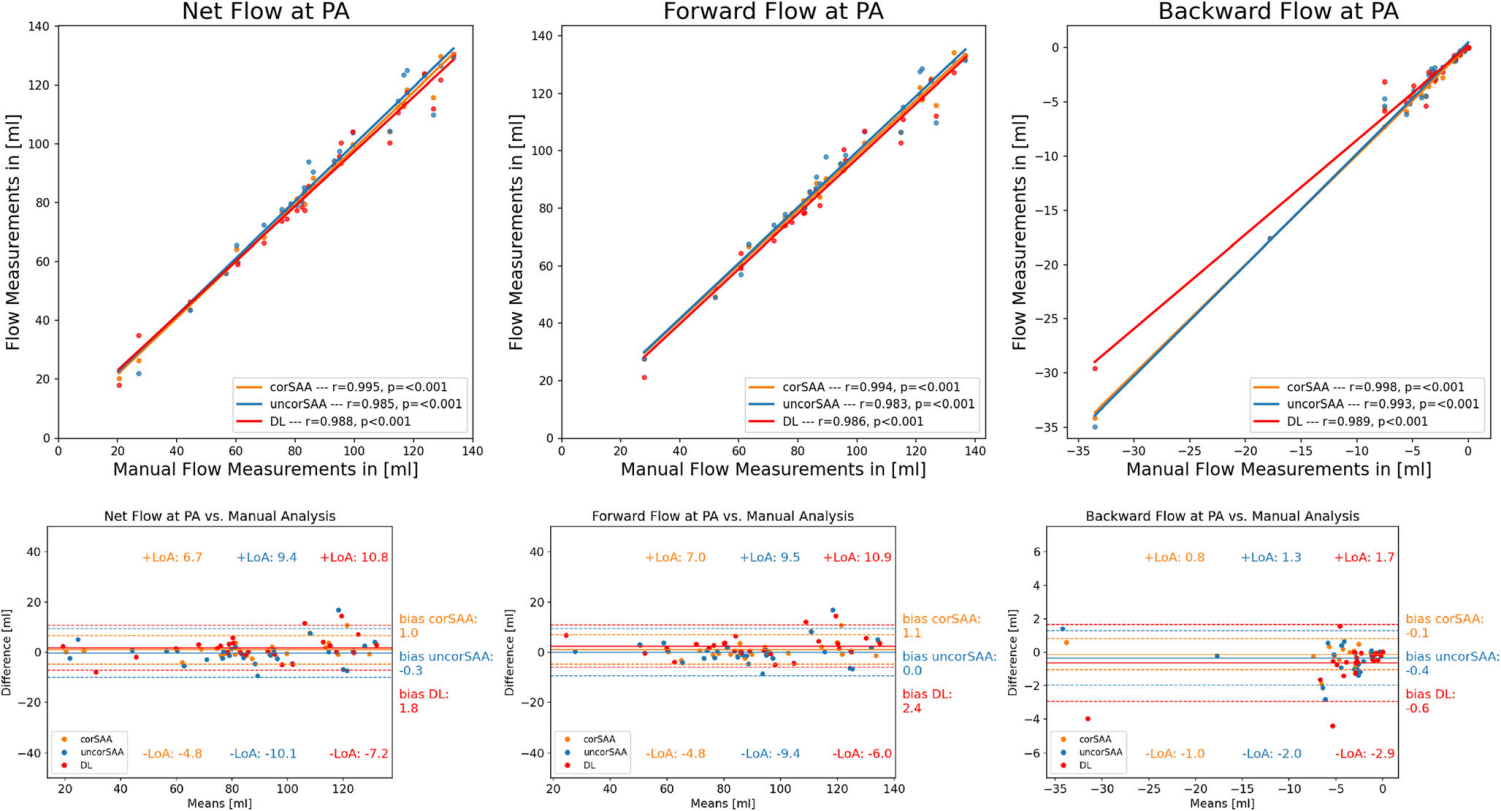
Flow Volume Measurements at PA. Comparison of net flow, forward flow, and backward flow at PA between manual analysis (MA, reference) with corrected semi-automated analysis (corSAA, orange), uncorrected semi-automated analysis (uncorSAA, blue), and deep learning (DL, red) analysis. Scatter plots (top) show excellent correlation and Bland-Altman plots (bottom) small bias with narrow limits of agreement (LoA) between human and DL measurements for each parameter while LoA were narrower compared to STJ. corSAA, corrected semi-automated analysis; DL, deep learning; PA, pulmonary artery; STJ, sinotubular junction; uncorSAA, uncorrected semi-automated analysis

At PA, the net flow was not significantly different between techniques (*p* = 0.051). However, forward flow differed significantly between the four techniques (*p* = 0.008). Compared to MA, DL analysis (bias = 2.4 mL, *p* = 0.009) reported lower values. Of note, DL analysis vs. corSAA were not significantly different (*p* = 0.08), similar to the results at STJ. Backward flow was significantly different between assessments (*p* = 0.006) which was caused by DL-analysis being lower than MA (bias = − 0.6 mL, *p* = 0.01) (Table 2, Figs. 3 and 4, and Supplement Tables 2, 3 (post hoc comparisons)).

#### Peak velocity

**Fig. 5:**
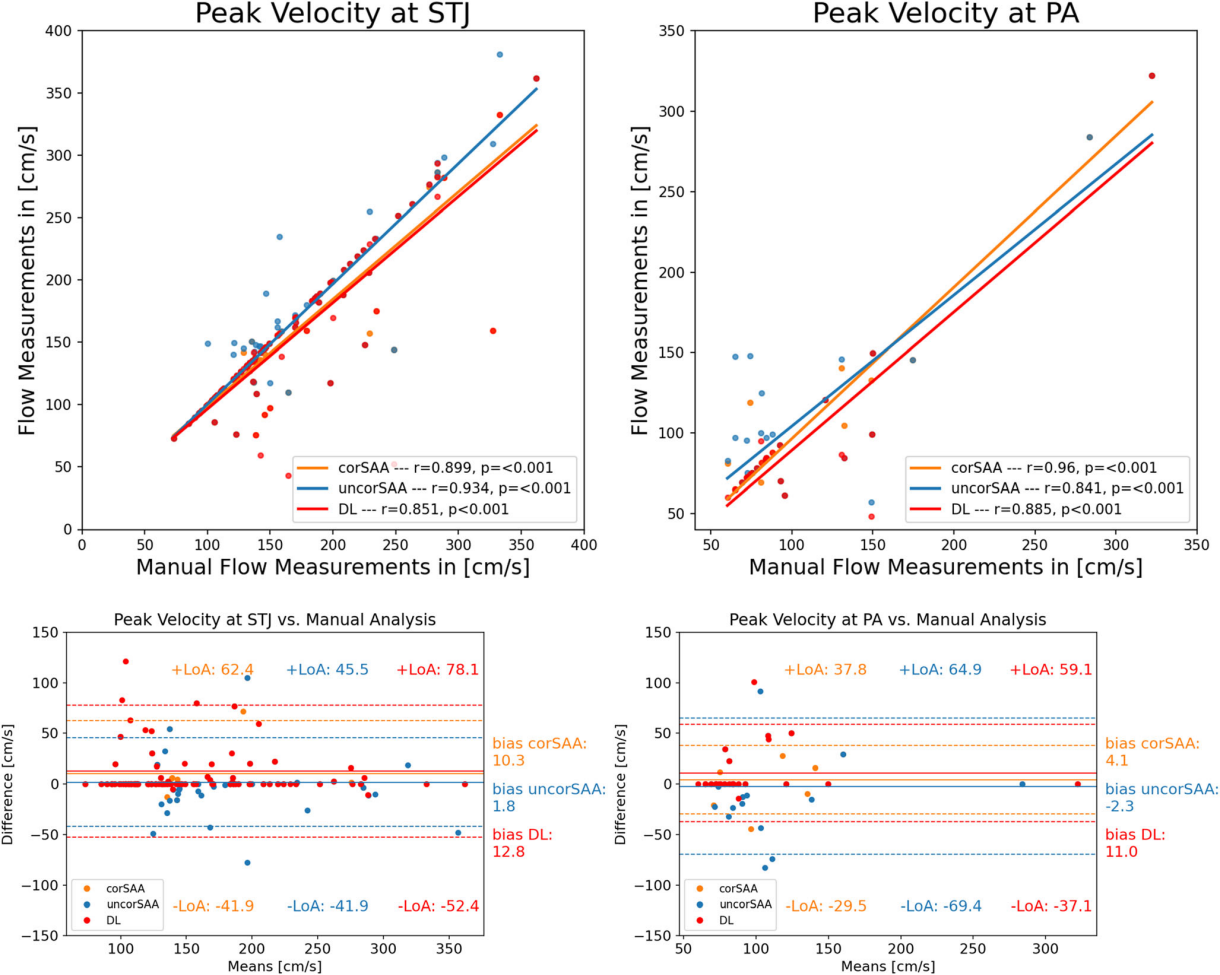
Flow velocity measurements at STJ and PA. Peak velocity comparison at STJ and PA between the manual analysis (MA, reference) with corrected semi-automated analysis (corSAA, orange), uncorrected semi-automated analysis (uncorSAA, blue) and deep learning (DL, red) analysis. Scatter plots (top) show overall excellent agreement at both locations; however, it is overall lower than for flow volume quantifications (see Figs. 3 and 4). corSAA, corrected smi-automated analysis; MA, manual analysis; PA, pulmonary artery; STJ, sinotubular junction; uncorSAA, uncorrected semi-automated analysis

At STJ, peak velocity analysis was significantly different between methods (*p* < 0.001). In post hoc tests, DL analysis (bias = 12.8 cm/s, *p* < 0.001) and corSAA (bias = 10.3 cm/s, *p* < 0.001) reported lower means compared to MA. Of note, DL analysis vs. corSAA assessment were not significantly different (*p* = 0.15). At PA, peak velocity was also significantly different between methods (*p* = 0.04). DL-analysis showed significantly lower means than MA (bias = 11.0 cm/s, *p* = 0.03) while compared to corSAA, the results were similar (*p* = 0.09) (Fig. 5; Table 2, Supplement Tables 2, 3 (post hoc comparisons)).

### Analysis of discrepant cases between DL and human assessment

**Fig. 6:**
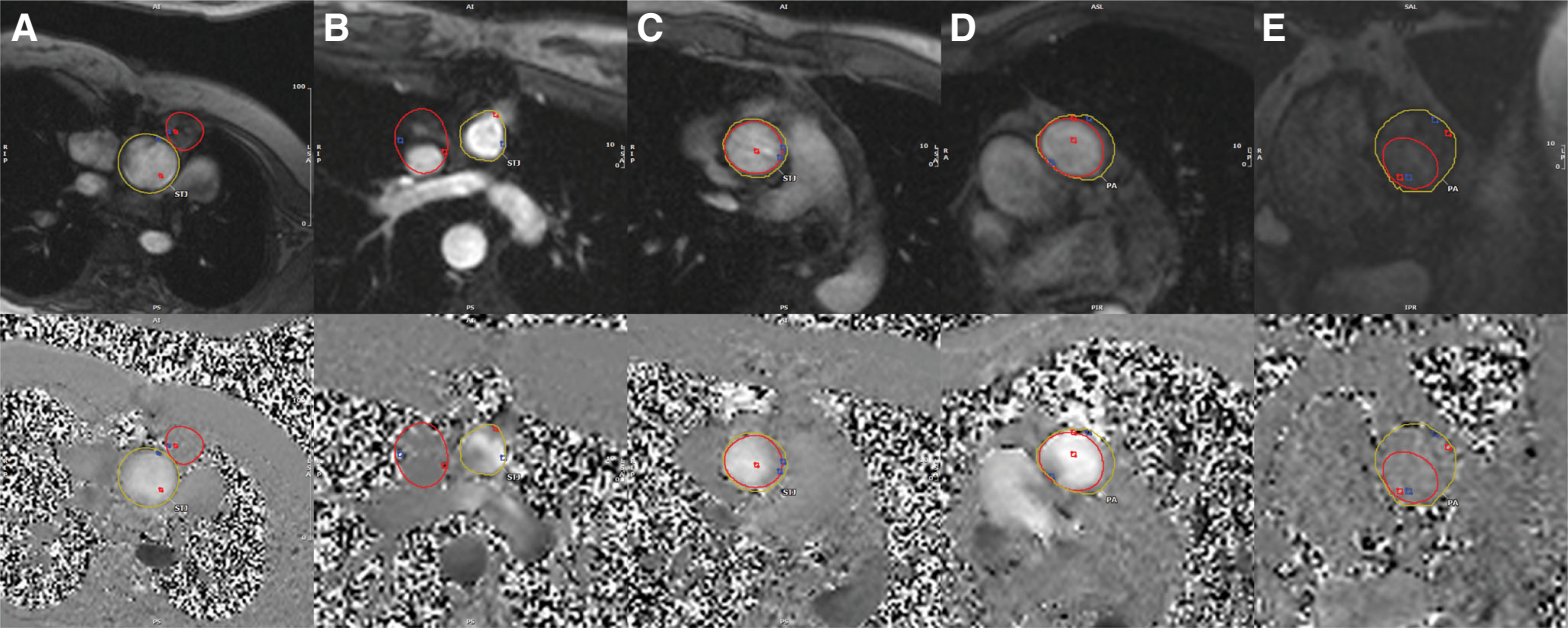
Erroneous cases. **A** The aorta was not segmented correctly on all cardiac time points by the DL tool leading to a mean DSC of 0.72 and a mean HD of 8.0 mm. This resulted in a difference in a net flow of 17 mL. **B** The vena cava superior was contoured by the DL tool (red contour); the aorta was delineated in MA by the CV radiologist. DSC was 0, and the difference in net flow was 51 mL. **C** The contour at STJ by the DL tool (red contour) was too small and did not cover the entire vessel. Thereby, the net flow was underestimated by 11 mL. **D** The contour by the DL at PA was too small during systole (red contour, DSC = 0.82 compared to MA), resulting in a net flow difference of 15 mL. **E** DSC was low during systole for PA, mainly caused by a small DL contour during one time point in early systole (DSC = 0.70). However, this did not result in a difference in flow parameters in this case. Notably, **A**, **B** were congenital heart disease patients. Red contour - DL tool, yellow contour - manual assessment. CV, cardiovascular; DL, deep learning; DSC, Dice score; HD, Hausdorff distance; MA, manual analysis; PA, pulmonary artery; STJ, sinotubular junction

Four cases showed a net flow difference of > ± 10 mL at the STJ and two at the PA (see examples in Fig. 6). Two of the STJ cases included patients with congenital heart disease with altered anatomy which resulted in partial contouring of the superior vena cava and the sternum by the DL tool, respectively. As a result, the net flow was reduced by 17 mL and 51 mL compared to MA and the DSC between MA and DL-analysis were 0.72 and 0, respectively (Fig. 6A, B). In two instances in both STJ and PA, the DL contours were too small, thereby missing the edge of the vessel, leading to an underestimation of net flow between 11 and 17 mL compared to MA (Fig. 6C, D).

A total of 29 cases showed a discrepancy in peak velocity > ± 10 cm/s. At STJ, 19/20 were false-high velocities in human assessment (16/20 MA, 3/20 corSAA) and 1/20 in DL analysis (incorrectly delineated contour, Fig. 6B). At PA, 8/9 cases (88.9%) were also caused by noisy voxels at the vessel’s border in the human measurements (5/9 MA, 4/9 corSAA, one case in both methods). DL analysis did not contour the vessel at every time point in the other case which resulted in the missing of the actual peak velocity. After removing outliers from peak velocity assessment which primarily affected MA measurements, MA vs. DL bias at STJ decreased to 2.8 cm/s (Pearson *r*: 0.99, *p < *0.001) and bias at PA decreased to 3.8 cm/s (Pearson *r*: 0.95, *p* < 0.001).

Overall, after a detailed review, 113/121 cases (93.4%) showed accurate measurements of net flow and peak velocity by the DL tool.

#### Analysis time

The DL tool performed a complete analysis in 0:01 ± 0 min per case. Compared to DL, MA took significantly longer at both STJ (mean: 3:00 ± 0:52 min, *p* < 0.001) and PA (mean: 2:47 ± 0:42 min, *p* < 0.001). In addition, corSAA also required significantly more time than DL-analysis at STJ (mean: 2:49 ± 1:15 min, *p* < 0.001) and PA (mean: 2:26 ± 1:19 min, *p* < 0.001).

### Inter-observer assessment

Comparison of manual contours between the CV radiologist and the resident was excellent at both STJ (DSC = 0.90 (IQR = 0.03) with small HD of 2.1 mm (IQR = 0.4 mm)) and at PA (DSC = 0.90 (IQR = 0.03) with HD of 1.9 mm (IQR = 0.2 mm)) between observers.

Flow parameters at STJ were not significantly different for net flow. However, forward flow (bias: 4.5 mL (*p* = 0.004), backward flow (bias: − 2.1 mL (*p* = 0.03) and peak velocity (bias: 7.3 cm/s, *p* = 0.02) showed small but significant differences (Table 3, Supplement Figure 2). At PA, all parameters showed small but statistically significant differences between the two readers. The biggest difference was seen for peak velocity assessment (bias = 13.4 cm/s, *p* = 0.04) which was impacted by three cases with noisy voxels at the vessels’ boundaries (Table 3, Supplement Figure 3).

**Table 3:** Inter-reader comparison at STJ and PA

	Reader 1	Reader 2	*p* value	Difference	Pearson r	Bias	Limits of agreement
*Sinotubular Junction*							
Net flow	88.7 ± 26.6 mL	86.4 ± 26.5 mL	0.105	2.6%	0.99 (*p *< 0.001)	2.4 mL	− 6.1–10.8 mL
Forward flow	106.4 ± 30.1 mL	101.9 ± 29.7 mL	**0.004**	4.2%	1.00 (*p *< 0.001)	4.5 mL	− 0.6−9.5 mL
Backward flow	− 17.7 ± 18.4 mL	− 15.5 ± 16.9 mL	**0.03**	12.4%	0.99 (*p *< 0.001)	− 2.1 mL	− 7.8–3.6 mL
Peak velocity	169.6 ± 58.0 cm/s	162.3 ± 58.3 cm/s	**0.02**	4.5%	0.96 (*p *< 0.001)	7.3 cm/s	− 24.4–39.04 cm/s
*Pulmonary Artery*							
Net flow	82.0 ± 34.5 mL	77.1 ± 30.6 mL	**0.04**	6.0%	0.99 (*p *< 0.001)	4.9 mL	− 5.3–15.0 mL
Forward flow	87.7 ± 28.7 mL	81.9 ± 26.0 mL	**0.01**	6.6%	0.99 (*p *< 0.001)	5.8 mL	− 2.8–14.3 mL
Backward flow	− 5.7 ± 10.1 mL	− 4.8 ± 8.9 mL	**0.008**	15.8%	1.00 (*p *< 0.001)	− 0.9 mL	− 3.1–1.3 mL
Peak velocity	93.5 ± 25.6 cm/s	80.2 ± 22.7 cm/s	**0.04**	13.3%	0.72 (*p *= 0.02)	13.4 cm/s	− 20.7–47.4 cm/s

Inter-reader comparison for MA revealed small but significant differences for all parameters at STJ and PA except net flow at STJ. Overall, the inter-reader differences were within the range of the MA vs. DL assessment. Significant *p* values are in boldface. *DL* deep learning, *PA* pulmonary artery, *SD* standard deviation, *STJ* sinotubular junction

## Discussion

We investigated the performance of a DL tool for fully-automatic location detection, contour delineation, and flow quantification of 2D-CINE-PC-MRI data at STJ and PA. The major results were: The evaluated DL tool showed a high technical success rate, analyzing the majority of included series (121/125, 97%) and identifying the respective location in all but four cases correctly. Furthermore, evaluation of net, forward and backward flow revealed that overall, there was excellent agreement at both locations between DL’s quantifications and human assessments (MA and corSAA) performed by a CV radiologist. Eventually, a detailed review of discrepant cases between human and DL measurements confirmed that more than 93% of DL measurements were accurate.

### General feasibility of the DL tool

Location detection in 2D-CINE-PC-MRI has been shown for the ascending and descending aorta in previous studies. However, imaging planes were acquired in axial orientation and not perpendicular to the blood flow axis at the level of the STJ which is the clinically relevant position [14, 15]. In addition, these studies only reported the contoured area but not the flow parameters. In 2014, shape detection for the analysis of the aorta was introduced which allowed reporting flow parameters but required a round shape as a target. While this might be feasible for the STJ, it is not optimal for the non-rounded shape of the aortic sinuses or the proximal PA [16]. Though the results in this prior study were comparable to ours in regard to bias, they were based on specific acquisition planes and were only available for the aorta [16]. Bratt et al presented a fully-automated deep learning method for the aorta; this tool is not generally available and it does not process the PA location [9]. The currently available software solutions for 2D-CINE-PC-MRI-based flow assessment are not fully-automatic and/or do not use deep learning for contour delineation. For example, the standard tools from some vendors, syngo.via (Siemens Healthineers) or segment (Medviso AB), require user input for example manually selecting the vessel or manual drawing of the initial contour which will then be forwarded automatically [17, 18]. To achieve reliable and reproducible results, contours need to be precise and accurate at all times points, thereby delineating the vessel boundaries to quantify all voxels with flow information within the vessel. If contours are too wide, flow calculations can be underestimated or image noise could affect results; excluding parts of the vessel will result in underestimation [2]. In our study, DSC were overall high but slightly higher at STJ compared to PA between MA by the experienced observer and DL tool. This variance at STJ was similar compared to inter-reader agreement, did not differ between systole and diastole, and was similar to the reports in the literature [9, 17]. At PA, DL vs. human agreement was slightly lower compared to inter-radiologists’ agreement (DSC: 0.85 vs. 0.90). Furthermore, DSC was lower in diastole, which was also found in another study [5]. The smaller PA training data set could explain that. 2D-CINE-PC-MRI assessment is generally more likely to be applied to STJ than PA assessment. We found that only 28.9% of patients (28/97) underwent PA assessment which is likely related to aortic (valve) pathologies being more common [16]. Our DSC results were impacted by few outlier cases, most of which were related to congenital heart disease cases with altered anatomy. Overall, a high technical success rate of 97% including reliable location detection as well as the high agreement of contours between human and DL assessment support the feasibility of the DL tool.

### Flow quantification

There are two major groups of flow parameters: flow volumes (forward, backward and net flow) and peak velocity. Depending on the respective situation, either parameter can be important for clinical classification. For example, if valve regurgitation is present, the backward volume is an important parameter. In the case of valve stenosis, peak velocity is of importance, and in the case of a cardiac shunt, determination of (systemic and pulmonary) net flows are important. Our analysis showed high consistency between the DL measurements and human assessments for forward, backward and net flow at both STJ and PA with the exception of four cases. Those results are similar to previous studies; however, those studies did not perform automatic location detection, did not use clinical series, or did not apply deep learning for segmentation [5, 9].

The results of peak velocity assessment showed more variance and studies on peak velocity assessment in the literature are limited. While Pearson correlation still showed excellent but relative to flow volume assessment lower agreement, we observed mean underestimation of peak velocity in DL-analysis compared to MA of −12.8 cm/s at STJ and of −11.0 cm/s at PA and similar numbers when compared to corSAA. However, a detailed analysis of the 29 cases with a discrepancy of > 10 cm/s between methods revealed that in most cases (27/29 cases, 93%), false-high velocities due to noisy voxels at the vessel’s border were reported in MA and corSAA assessments. Only in two cases, the peak velocities reported by the DL tool were inaccurate. This highlights the importance of accurate contour delineation and one advantage of DL tool which reported peak velocity more reliable than MA and corSAA. Peak velocity assessment by corSAA was more reliable than MA which could be related to MA being more cumbersome. Since peak velocity assessment is based on a single voxel value in the software used for this study, increasing the region of interest (ROI) could be considered, for example from 1 × 1 to 2 × 2 voxels. However, this would reduce the measured peak velocity (the mean of these voxels is reported ). We think that this is not generally feasible, especially in situations like (aortic) valve stenosis where peak velocity is an important marker to grade the level of valve disease [19]. None of the studies investigating semi-automatic vessel contouring reported peak velocities which highlights an advantage of this DL tool [5, 9]. In total, 93.4% of processed cases were accurate by the DL tool in regard to comprehensive flow assessment. Adjustments would have only been necessary in eight outlier cases (6.6%), most of which were congenital heart disease cases. Another advantage of the DL tool derived from inter-observer assessment: Comparing the CV radiologist and a CV-trained resident showed overall good to excellent agreement at STJ and PA. However, there were slight differences. The DL tool showed zero variability in repeated measurements while bias and limits of agreements were in the same range compared to the MA and the corSAA methods by the CV radiologist. This indicates that the DL tool can help to reduce measurement variability. Furthermore, measurement times for MA and corSAA were about 3 minutes per case and location which could be saved in the vast majority of cases using DL-analysis which highlights another strength of the DL tool.

Interestingly, we did find smaller differences in contours and flow parameters between the uncorSAA compared to corSAA and MA than initially expected. However, planes without adjustments equal to a DSC of 1.0 (and HD of 0.0 mm) skewed these results [2].

Limitations of our study are the single center and retrospective approach. In addition, we only analyzed 2D-CINE-PC series from one vendor. We analyzed consecutive patients who underwent 2D-CINE-PC-MRI with different indications for the scan. This heterogeneity could have influenced our results; hence, we included all patients from a single month (November 2020) in order to get a representative cohort. Only about 30% of cases in this cohort had 2D-CINE-PC-MRI for the PA but all had an assessment of the aorta. While ideally, all patients should have had both locations available, this was also related to our institutional clinical cohort for which there is generally less often an indication to assess PA flow.

In summary, the evaluated DL tool performed fully-automated location detection and flow quantification of the aorta and pulmonary artery in more than 93% of cases at the expert level. Because of few outlier cases, a visual inspection must be performed, however, with results being available instantaneously, our data suggests that this DL tool could be integrated seamlessly and increase efficiency in the clinical workflow for assessment of 2D-CINE-PC-MRI.

## Supplementary information

ESM 1 (DOCX 807 kb)

## Abbreviations

2D: 2-dimensional
4D: 4-dimensional
AI: Artificial intelligence
corSAA: Corrected semi-automated analysis
CV: Cardiovascular
DL: Deep learning
DSC: Dice similarity coefficient
HD: Hausdorff distance
IQR: Interquartile range
LoA: Limits of agreement
LV: Left ventricle
MA: Manual analysis
MRI: Magnetic resonance imaging
PA: Pulmonary artery
PC: Phase contrast
ROI: Region of interest
STJ: Sinotubular junction
TE: Echo time
TR: Repetition time
uncorSAA: Uncorrected semi-automated analysis
VENC: Velocity encoding

## Declarations

### Guarantor

The scientific guarantor of this publication is Maurice Pradella.

### Statistics and biometry

No complex statistical methods were necessary for this paper.

### Methodology

• retrospective

• diagnostic study

• performed at one institution
