# Supplementary material for: Fully-automated deep learning-based flow quantification of 2D CINE phase contrast MRI
Source: Eur Radiol. 2022 Oct 29;33(3):1707–18. doi: 10.1007/s00330-022-09179-3 (PMC9935671; doi:10.1007/s00330-022-09179-3)
Supplement: Supplementary file 1 — (DOCX 807 kb) [file 330_2022_9179_MOESM1_ESM.docx]

# Supplements

## Deep Learning Algorithm

### Methodology

The DL tool was built as a two-stage model, performing classification and segmentation. Classification of series types was based on the flow series image orientation information, which is a 3-dimensional normal vector perpendicular to the acquisition plane. Since both aortic and PA types have their characteristic image acquisition planes, a deep convolutional neural network (DCNN) model was trained to predict series type based on the input image orientation from either of the aortic, pulmonic, and other flow series types. The input to the classification network is a known image orientation information. A fully connected network with two hidden layers is developed with a softmax termination layer which outputs class probabilities corresponding to three desired output classes (aortic, pulmonic, or other). The segmentation stage involved two networks trained specifically to perform contouring corresponding to the type of flow series. This ensured models to learn different anatomies (aortic and pulmonic location) and that the respective vessel boundaries were contoured. The segmentation model performed binary segmentation task, where for a given input image a labelled image (mask) was output in which the vessels’ pixels were labelled as 1 (foreground) while the remaining pixels are labelled as 0 (background).

### Network Architecture

Deep convolutional neural network architectures were used for both series classification and segmentation tasks. Network training was conducted using the TensorFlow framework on a 12 GB RAM Nvidia Titan Xp graphics card (Nvidia Corporation). Best epochs were selected based on the validation performance. Training was performed on internal data and data from the UK Biobank [SUP_1], see paragraph Training below.

Classification

The input to the classification network is a known image orientation information. A fully connected network with two hidden layers is developed with a termination layer of softmax to outputs class probabilities corresponding to three desired outputs classes.

Segmentation

Both aortic and pulmonic segmentation networks use a U-Net style architecture [SUP_2]. Each network inputs a magnitude image as an input and outputs a binary mask representing the region of interest (aortic lumen or pulmonary artery). This architecture features an encoder-decoder structure, and long-range shuttle connections that facilitate the gradient flow and improved usage of the low-level details. A batch normalization layer was inserted before the U-Net to standardize the intensity of the input [SUP_3]. The final layer is a softmax layer that outputs the probabilities of each pixel being a valve (foreground) or background. The inputs were resampled and center cropped to a shape of 192x192 pixels with a pixel size of 1.855x1.855 mm/pixel using a spatial transformer [SUP_4]. A reverse transform was applied to convert the segmentation output back to the original image size.

### Training

#### Training Data sets

Data from multiple sources was collected to perform models training. Majority of the data was sourced from the UK BioBank (UKBB) database [SUP_1], which included only aortic type data. Pulmonic artery type data was used from internal data sets (Circle Cardiovascular Imaging) that included exams from research collaborations. A breakdown of training data is included in the Supplement Table 1.

Supplement Table 1: Training data sources, types, and number of cases

| **Data set** | **Type** | **No. of images** | **Task** |
| --- | --- | --- | --- |
| D1: Internal data set | aorta, pulmonic artery | 1881 (aorta: 1177, pulmonic artery: 197, Others: 507) | aortic and pulmonic classification |
| D2: UKBB | aorta | 23550 | aortic segmentation |
| D3: Internal data set | pulmonic artery | 1065 | pulmonic segmentation |

#### Classification

The training data for series classification task included data set D1 as shown in Supplement Table 1. A total of 1881 flow images were used for this task with a training and testing split of 75% and 25%, respectively. The ground truth data for this task was prepared using known input image orientation and output series types. Three class labels including 0, 1, and 2 were defined based on type of flow image from either aortic, pulmonic, or other category.

#### Segmentation

### For the segmentation task, multiple data sets were used. The aortic type data came from data source D2 and was split into training and testing sets with a ratio of 80% to 20%, respectively. Whereas the pulmonic type data came from D3 source, which was also split into training and validation sets with 80% and 20% ratios, respectively (Supplement Table 1). The ground truth data for the segmentation tasks involved manual contouring (STJ and PA) by a radiologist with a predefined standard operating procedure (SOP) aortic and pulmonic type images are shown in Supplement Fig. 1. Implementation Details

#### Classification

Cross-entropy loss and F1 loss with equal weighting were used to train the classifier. An Adam optimizer with beta values of 0.9 and 0.999 was used during training [13]. The batch size and learning rate were set to 100 and 0.0001, respectively.

#### Segmentation

Cross-entropy loss and Dice loss with equal weighting were used to train both segmentation networks. Adam optimizer with the same setting as in the classification was used. The batch size and learning rate were set to 64 and 0.0001, respectively. Typical image augmentation techniques, including mirroring, rotation, zooming, gamma transform, histogram equalization, and cutout were applied randomly with a pre-set probability to improve the network generalizability specially on PA type images for which less training data was available (STJ: 23550 cases vs. PA 1065 cases, see Supplements) [14]. In addition, a geometric mask of a rectangular or circular shape was also randomly applied to mask out the peripheral background region to mimic MR images seen in clinical practice.


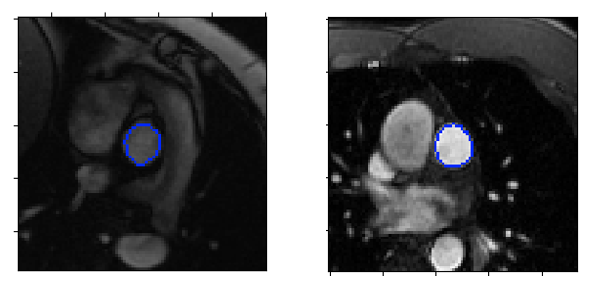


Supplement Figure 1. Exemplar magnitude images, (left) aortic series type (right) pulmonic series type.

## Results

DL Performance for Vessel Contour Delineation


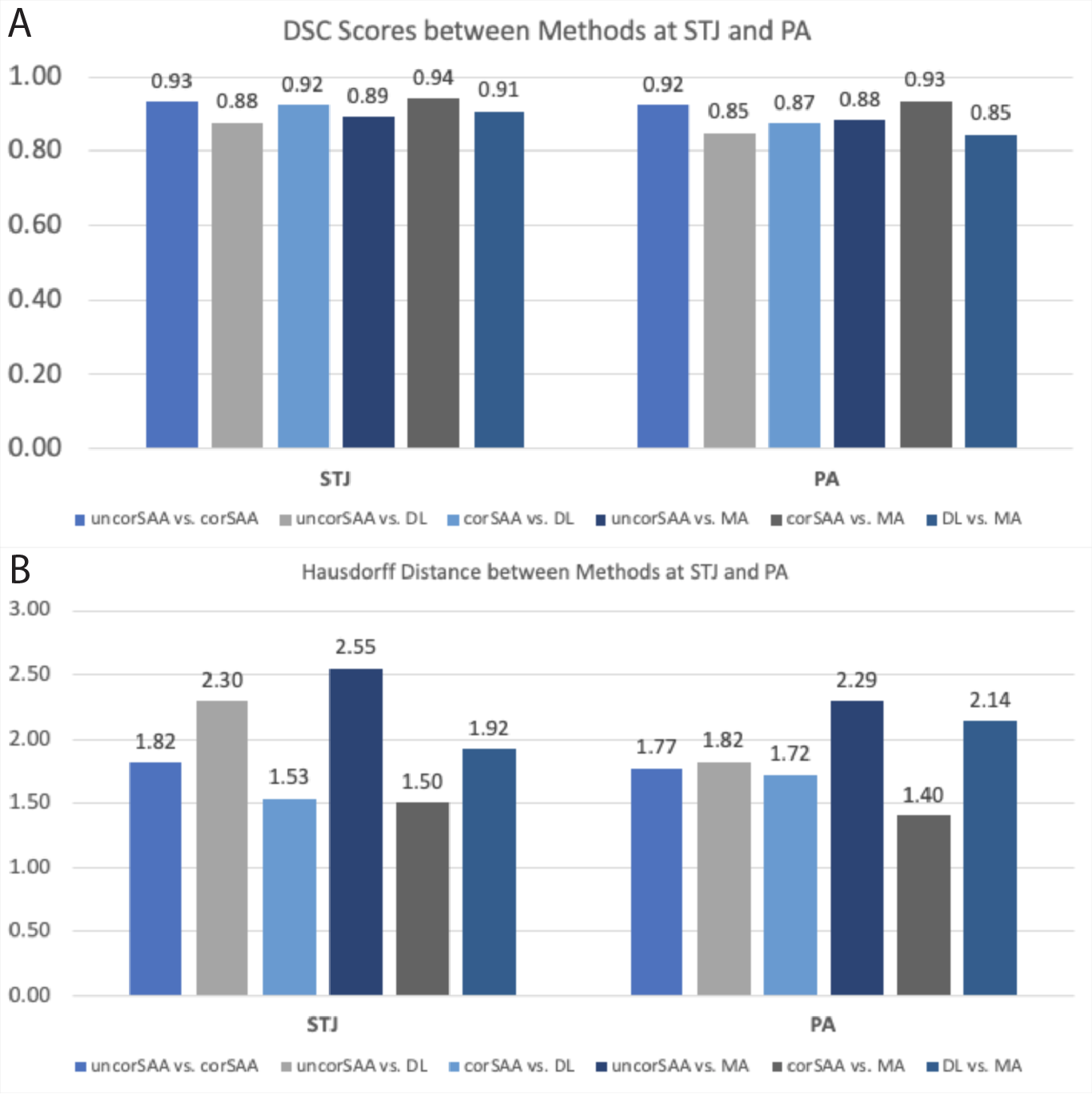


Supplement Figure 2. Comparisons for DSC scores (A) and Hausdorff distances (B) between contouring techniques.

### Posthoc comparisons of Flow Analysis

Supplement Table 2: Sinotubular Junction

| **Parameter A** | **Parameter B** | **Paired** | **Parametric** | **T** | **dof** | **alternative** | **p value** | **BF10** | **hedges** |
| --- | --- | --- | --- | --- | --- | --- | --- | --- | --- |
| Forward Flow | | | | | | | | | |
| DL | corSAA | TRUE | TRUE | -1.925533 | 94 | two-sided | 0.057185 | 0.665 | -0.042527 |
| DL | MA | TRUE | TRUE | -2.60324 | 94 | two-sided | 0.010732 | 2.737 | -0.059231 |
| DL | uncorSAA | TRUE | TRUE | -1.692035 | 94 | two-sided | 0.093952 | 0.447 | -0.047462 |
| corSAA | MA | TRUE | TRUE | -2.169022 | 94 | two-sided | 0.032604 | 1.058 | -0.017119 |
| corSAA | uncorSAA | TRUE | TRUE | -0.359617 | 94 | two-sided | 0.71994 | 0.121 | -0.005341 |
| MA | uncorSAA | TRUE | TRUE | 0.730135 | 94 | two-sided | 0.467124 | 0.147 | 0.011664 |
| Backward Flow | | | | | | | | | |
| DL | corSAA | TRUE | TRUE | 3.295603 | 94 | two-sided | 0.001386 | 16.87 | 0.039251 |
| DL | MA | TRUE | TRUE | 5.132415 | 94 | two-sided | 0.000002 | 9329.949 | 0.076286 |
| DL | uncorSAA | TRUE | TRUE | 2.871329 | 94 | two-sided | 0.005051 | 5.305 | 0.056821 |
| corSAA | MA | TRUE | TRUE | 2.982736 | 94 | two-sided | 0.003639 | 7.097 | 0.038108 |
| corSAA | uncorSAA | TRUE | TRUE | 1.273452 | 94 | two-sided | 0.205998 | 0.248 | 0.01903 |
| MA | uncorSAA | TRUE | TRUE | -1.174328 | 94 | two-sided | 0.24323 | 0.221 | -0.018229 |
| Peak Velocity | | | | | | | | | |
| DL | corSAA | TRUE | TRUE | -1.431415 | 94 | two-sided | 0.155628 | 0.304 | -0.040543 |
| DL | MA | TRUE | TRUE | -3.736878 | 94 | two-sided | 0.00032 | 64.182 | -0.208725 |
| DL | uncorSAA | TRUE | TRUE | -4.160939 | 94 | two-sided | 0.00007 | 260.397 | -0.175145 |
| corSAA | MA | TRUE | TRUE | -3.748013 | 94 | two-sided | 0.000308 | 66.493 | -0.172783 |
| corSAA | uncorSAA | TRUE | TRUE | -3.523191 | 94 | two-sided | 0.00066 | 33.066 | -0.139029 |
| MA | uncorSAA | TRUE | TRUE | 0.810549 | 94 | two-sided | 0.419672 | 0.156 | 0.030126 |

Supplement Table 3: Pulmonary Artery

| **Parameter A** | **Parameter B** | **Paired** | **Parametric** | **T** | **dof** | **alternative** | **P value** | **BF10** | **hedges** |
| --- | --- | --- | --- | --- | --- | --- | --- | --- | --- |
| Forward Flow | | | | | | | | | |
| DL | corSAA | TRUE | TRUE | -1.855812 | 25 | two-sided | 0.075307 | 0.917 | -0.044811 |
| DL | MA | TRUE | TRUE | -2.837931 | 25 | two-sided | 0.008881 | 5.217 | -0.091672 |
| DL | uncorSAA | TRUE | TRUE | -2.736834 | 25 | two-sided | 0.011254 | 4.274 | -0.086783 |
| corSAA | MA | TRUE | TRUE | -2.129831 | 25 | two-sided | 0.043211 | 1.418 | -0.047697 |
| corSAA | uncorSAA | TRUE | TRUE | -1.650159 | 25 | two-sided | 0.11142 | 0.682 | -0.042575 |
| MA | uncorSAA | TRUE | TRUE | 0.144117 | 25 | two-sided | 0.886564 | 0.209 | 0.005278 |
| Backward Flow | | | | | | | | | |
| DL | corSAA | TRUE | TRUE | 2.358651 | 25 | two-sided | 0 | 2.105 | 0.089037 |
| DL | MA | TRUE | TRUE | 2.725103 | 25 | two-sided | 0.011565 | 4.177 | 0.103266 |
| DL | uncorSAA | TRUE | TRUE | 1.390007 | 25 | two-sided | 0.176781 | 0.488 | 0.051056 |
| corSAA | MA | TRUE | TRUE | 0.885009 | 25 | two-sided | 0.384585 | 0.296 | 0.012727 |
| corSAA | uncorSAA | TRUE | TRUE | -1.860678 | 25 | two-sided | 0.074592 | 0.924 | -0.034554 |
| MA | uncorSAA | TRUE | TRUE | -1.900636 | 25 | two-sided | 0.06894 | 0.982 | -0.047344 |
| Peak Velocity | | | | | | | | | |
| DL | corSAA | TRUE | TRUE | -1.772696 | 25 | two-sided | 0.088466 | 0.811 | -0.145887 |
| DL | MA | TRUE | TRUE | -2.238321 | 25 | two-sided | 0.034345 | 1.704 | -0.207681 |
| DL | uncorSAA | TRUE | TRUE | -3.052889 | 25 | two-sided | 0.005316 | 8.069 | -0.277096 |
| corSAA | MA | TRUE | TRUE | -0.97813 | 25 | two-sided | 0.337381 | 0.319 | -0.063002 |
| corSAA | uncorSAA | TRUE | TRUE | -1.335079 | 25 | two-sided | 0.193881 | 0.458 | -0.130957 |
| MA | uncorSAA | TRUE | TRUE | -0.508431 | 25 | two-sided | 0.615609 | 0.233 | -0.066781 |

### Inter-observer assessment

The CV radiologist was significantly faster than the resident at both location in the order of 6 minutes per location (STJ mean: 2:58±1:01min vs. 9:46±3:41min, p<0.001 and PA mean: 2:52±0:44min vs. 9:45±3:02min, p<0.001).


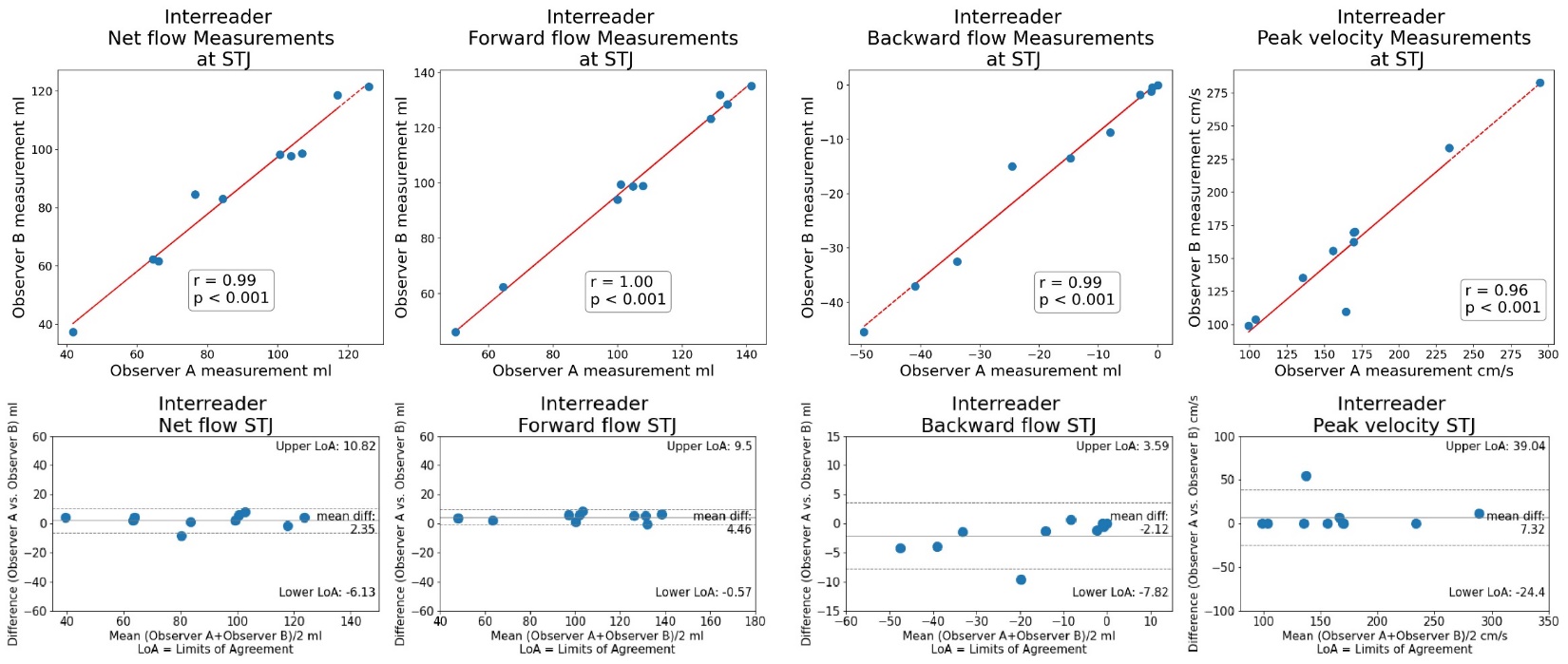


Supplement Figure 2: Inter-observer assessment at STJ for net flow, forward flow, backward flow, peak velocity. Agreement was excellent overall, however, bias for each parameter was slightly higher compared to the DL algorithm.

DL – Deep learning, STJ – Sinotubular junction


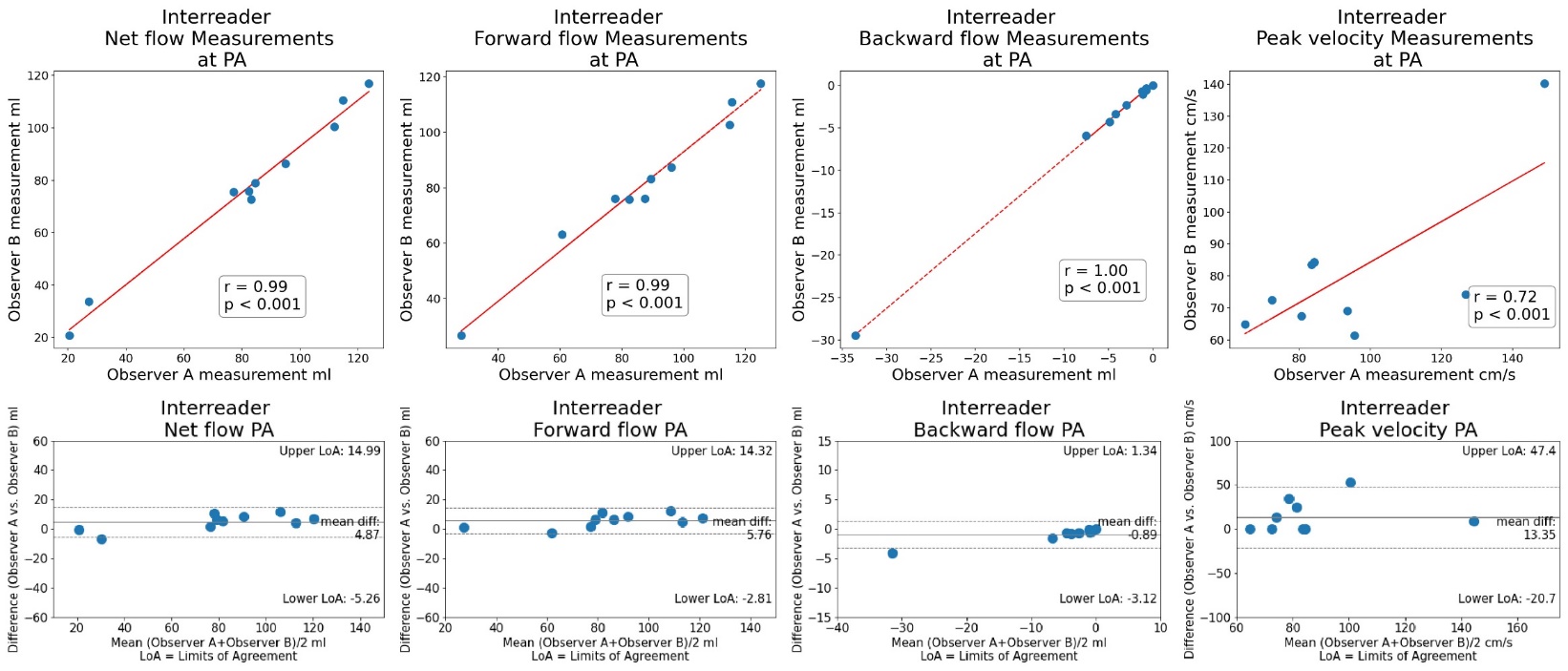


Supplement Figure 3: Inter-observer assessment at PA for net flow, forward flow, backward flow, peak velocity. For net flow, forward and backward flow, agreement was excellent as seen at STJ. Peak velocity showed good correlation which was caused by three outliers in the CV radiologist’s assessment (noisy voxel).

CV – Cardiovascular, PA – Pulmonary artery, STJ – Sinotubular junction

## References Supplement

[SUP_1] Sudlow, Cathie, et al. "UK biobank: an open access resource for identifying the causes of a wide range of complex diseases of middle and old age." *PLoS medicine* 12.3 (2015): e1001779.

[SUP_2] Ronneberger O, Fischer P, Brox T (2015) U-Net: Convolutional Networks for Biomedical Image Segmentation. CoRR abs/1505.04597

[SUP_3] Ioffe S, Szegedy C (2015) Batch Normalization: Accelerating Deep Network Training by Reducing Internal Covariate Shift. CoRR abs/1502.03167

[SUP 4] Jaderberg M, Simonyan K, Zisserman A, Kavukcuoglu K (2015) Spatial Transformer Networks. CoRR abs/1506.02025
